# Adjuvant Radiotherapy Following Surgical Excision of Keloids: A Systematic Review of Dose, Fractionation, and Recurrence

**DOI:** 10.3390/life16050770

**Published:** 2026-05-03

**Authors:** Monika Wojarska, Klaudia Kokot, Wiktoria Borzyszkowska, Patryk Boczar, Zuzanna Zalewska, Adrianna Kuryk, Julia Wojciechowska, Jerzy Jankau

**Affiliations:** 1 Plastic Surgery Clinic, Medical University of Gdańsk, Smoluchowskiego, 80-214 Gdańsk, Poland; jerzy.jankau@gumed.edu.pl; 2 Students’ Scientific Circle of Plastic Surgery, Plastic Surgery Department, Medical University of Gdańsk, 80-210 Gdańsk, Poland; wborzyszkowska@gumed.edu.pl (W.B.); patrykboczar@gumed.edu.pl (P.B.); zuzanna.zalewska@gumed.edu.pl (Z.Z.); kurykadrianna@gumed.edu.pl (A.K.); julwoj@gumed.edu.pl (J.W.); 3 Scientific Circle of Neurotraumatology, Department of Emergency Medicine, Medical University of Gdańsk, 80-210 Gdańsk, Poland

**Keywords:** radiotherapy, brachytherapy, keloids, surgical excision, systematic review

## Abstract

Keloids are pathological scars originating from connective tissue characterized by excessive growth that extends beyond the original edges of the wound. They occur significantly more often in skin areas exposed to increased mechanical tension during the wound-healing process and up to fifteen times more frequently in individuals with darker skin pigmentation. The underlying mechanism of keloid formation is driven by an inflammatory response triggered by skin injury extending into the reticular dermis, leading to fibroblast accumulation and neovascularization. The management of keloids remains challenging, as the recurrence rate is high when surgical excision is performed as a standalone treatment. Evidence indicates that combining surgical resection with adjunctive modalities results in superior clinical outcomes and may significantly lower recurrence rates compared with monotherapy. Adjuvant radiotherapy plays a key role in this approach, as it has been shown to reduce recurrence rates to below 10%, primarily through suppression of inflammation and inhibition of fibroblast activity. This systematic review adhered to the Preferred Reporting Items for Systematic Reviews and Meta-Analysis (PRISMA) guidelines. A systematic search of the PubMed and Web of Science databases identified 22 studies comprising more than 2219 patients treated with surgical excision followed by postoperative radiotherapy. Reported recurrence rates ranged from 1.6% to 55.2% and were influenced by total radiation dose, fractionation schedule, radiotherapy technique, and duration of follow-up. Despite its proven effectiveness when combined with surgery, radiotherapy has certain limitations, including the lack of standardized guidelines regarding dose, fractionation, and timing of administration. Most reported adverse effects were mild to moderate and localized to the treated area, while a direct causal relationship between postoperative radiotherapy and secondary malignancy development could not be established. The variability in treatment protocols highlights the need for further studies to support more effective, evidence-based decision-making in the treatment of keloids.

## 1. Introduction

Keloids are pathological scars originating from connective tissue. They are characterized by excessive growth that extends beyond the original edges of the wound [1]. The most common locations include the earlobe, mandibular margin, suprapubic region and the anterior chest. Keloids occur significantly more often in skin areas exposed to increased mechanical tension during the wound-healing process [2].

The underlying mechanism of keloid formation is driven by an inflammatory response triggered by skin injury extending into the reticular dermis. This damage leads to the accumulation of inflammatory cells and fibroblasts, accompanied by neovascularization within the affected tissue. When exacerbating factors—such as local mechanical stress, infection, or chronic irritation—prolong the inflammatory response, the process becomes chronic, ultimately contributing to pathological scar formation [3].

Mechanical tension plays a key role in the pathogenesis of keloids. Mechanical stimuli promote the activation of mechanotransduction pathways in fibroblasts that lead to upregulation of transforming growth factor-beta (TGF-β). Activation of the TGF-β/Smad signaling pathway leads to enhanced fibroblast proliferation and stimulates excessive extracellular matrix production [4].

Epidemiological data indicate that keloids occur significantly more frequently in individuals with darker skin pigmentation compared to those with lighter skin [5]. A hereditary component has also been demonstrated, as these pathological scars frequently exhibit familial clustering [6].

Keloids frequently present with symptoms such as pain, itching, and discomfort, all of which may negatively impact patients’ quality of life [7]. They may also result in visible disfigurement, adversely affecting psychosocial well-being. Moreover, when lesions occur in proximity to joints, they can restrict range of motion and contribute to functional impairment and reduced mobility [8].

The management of keloids remains challenging, as the recurrence rate is high when surgical excision is performed as a standalone treatment; therefore, therapeutic strategies should focus on inhibiting dermal inflammation rather than solely excising the lesion. Evidence indicates that combining surgical resection with adjuvant radiotherapy results in superior clinical outcomes; as has been shown, it may reduce recurrence rates to below 10% [9,10]. Some studies indicate that combination therapy involving radiotherapy may significantly reduce keloid recurrence compared to monotherapy. A meta-analysis of randomized controlled trials showed that a combination of triamcinolone and 5-fluorouracil with brachytherapy results in notably lower recurrence rates than intralesional steroid injection alone, which highlights that radiotherapy may be beneficial when added to a treatment regime [11]. Furthermore, studies confirm that radiotherapy performed after surgical excision can reduce recurrence rates to approximately 14–18% [12].

Therapies combined with surgery include radiotherapy, steroids injections, laser therapy, and compression therapy [13,14].

The effectiveness of radiotherapy is attributed to reduced neovascularization and a decrease in the supply of pro-inflammatory cytokines, which subsequently suppresses inflammation within the reticular dermis and inhibits fibroblast activity—processes central to keloid pathophysiology [15]. The therapeutic effect of radiotherapy results from both cytotoxic and molecular mechanisms. Ionizing radiation damages fibroblast DNA, generates reactive oxygen species (ROS) and disrupts cell cycle progression, further suppressing fibroblast proliferation which leads to the induction of apoptosis. Radiotherapy can also suppress the expression of TGF-β, reducing extracellular matrix production and collagen synthesis [16,17].

Available radiotherapy methods include electron beam radiotherapy, proton radiotherapy and brachytherapy. Clinical studies report recurrence rates after brachytherapy ranging from 4.9% to 24.1%, while recurrence rates after electron beam radiotherapy range from 1.6% to 55.2%.

Despite the available literature on adjuvant radiotherapy for keloid management, there is a lack of standardized treatment protocols. Therefore, this review aims to synthesize current evidence regarding radiation dose, fractionation protocols, associated adverse effects, proposed mechanisms of action, and reported recurrence rates. The objective is to inform clinical practice and support more effective, evidence-based decision-making in the treatment of keloids with the aim of establishing radiotherapy strategies that could lead to reducing keloid recurrence rates.

## 2. Materials and Methods

This study adhered to the Preferred Reporting Items for Systematic Reviews and Meta-Analysis (PRISMA) guidelines. A systematic search of the PubMed and Web of Science databases for articles published between January 2013 and April 2025 was performed using search terms designed to identify studies. A comparable search strategy was employed, using appropriate keywords and Boolean operators: ((radiotherapy) OR (brachytherapy)) AND (keloids) OR keloids radiotherapy OR keloids brachytherapy. The study has been registered in PROSPERO under registration number CRD420261336277.

Unrelated articles that were discovered by keyword matching were also excluded. Abstracts, case reports, conference papers, letters, editorials, and articles written in languages other than English were excluded during the initial screening of titles and citations. Studies duplicated in databases were removed using Mendeley Software (Reference Manager Version 2.130.2). After the initial search was performed by one researcher, duplicate records were removed. Two independent reviewers screened the remaining titles and abstracts according to predefined inclusion and exclusion criteria. Based on this screening process, potentially relevant full-text articles were identified and retrieved. Full-text articles were then independently assessed by the same two reviewers to determine whether they focused on radiotherapy on keloids. Disagreements at any stage were resolved by consensus. Final decisions were made under the supervision of the first author. Outcomes included keloid recurrence, defined as the reappearance of clinically evident scar tissue at the treated site during follow-up, time to recurrence rate, and patient-reported outcomes such as pain, pruritus, and cosmetic satisfaction, as reported in the included studies.

The eligibility criteria were defined according to the PICOS framework:

- Population (P): Patients with keloids undergoing surgical excision.
- Intervention (I): Postoperative radiotherapy (any modality).
- Comparison (C): Not consistently applicable due the observational nature of the included studies.
- Outcomes (O): Recurrence rate, time to recurrence, complications, and patient-reported outcomes.
- Study design (S): Cohort studies and clinical studies.

Exclusion criteria included case reports, reviews, conference abstracts, non-English articles, and studies without postoperative radiotherapy.

Of the 653 records initially identified through database searching (PubMed: n = 376; Web of Science: n = 277), 232 duplicates were removed, leaving 421 records for title and abstract screening.

During the screening process, 386 records were excluded based on title and abstract as they did not meet the inclusion criteria. The remaining 35 articles were considered potentially eligible and were retrieved for full-text assessment.

Following full-text review, 13 articles were excluded for the following reasons: a lack of postoperative radiotherapy, the absence of surgical excision of the keloid, or insufficient or irrelevant outcome data.

Ultimately, 22 studies met all inclusion criteria and were included in the qualitative synthesis.

**Figure 1:**
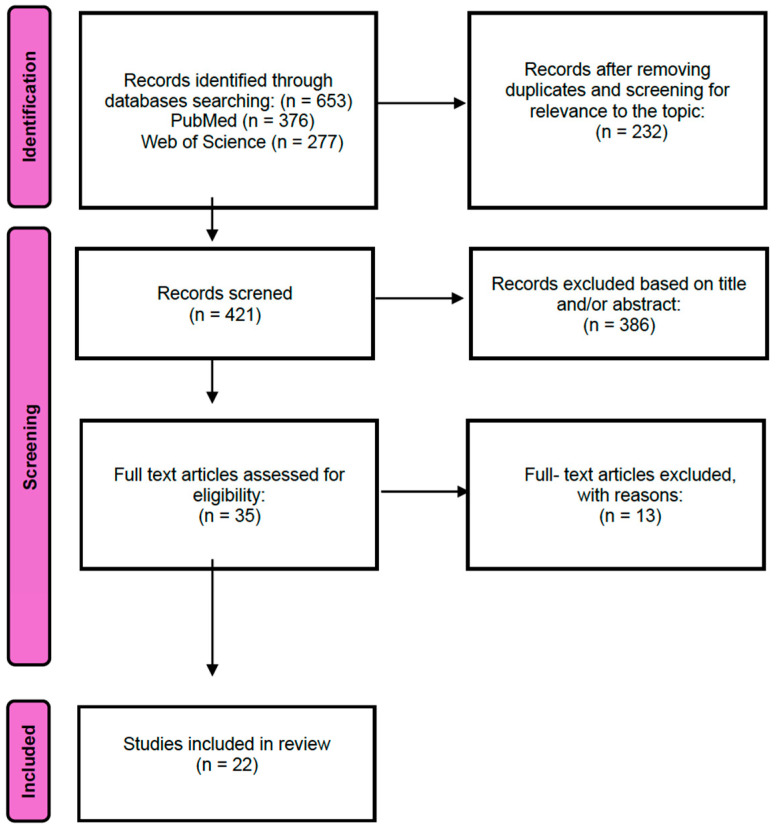
Prisma flowchart.

The study selection process is summarized in the PRISMA flow diagram (Figure 1), which details the number of records identified, screened, excluded, and included at each stage of the review.

The methodological quality and risk of bias of the included studies were independently assessed by two reviewers using the Newcastle–Ottawa Scale (NOS) for cohort studies. This tool evaluates studies across three domains: the selection of study groups (maximum 4 stars), comparability of groups (maximum 2 stars), and assessment of outcomes (maximum 3 stars), with a maximum total score of 9 stars.

Studies were categorized as high quality (7–9 stars), moderate quality (4–6 stars), or low quality (0–3 stars). Any discrepancies between reviewers were resolved through discussion and consensus.

The NOS scores were additionally used to assess the overall risk of bias. Studies with higher scores (≥7 stars) were considered to have a low risk of bias, while those with moderate scores (4–6 stars) were considered to have a moderate risk of bias, primarily due to limitations in comparability and potential confounding factors. No studies were classified as having a high risk of bias.

The results of the quality assessment are presented in Table 1 and were taken into account in the interpretation of the findings.

Due to substantial clinical and methodological heterogeneity among the included studies, a quantitative meta-analysis was not performed. The heterogeneity was primarily related to differences in radiotherapy techniques, total radiation dose, fractionation schedules, timing of administration, follow-up duration, and outcome reporting.

Instead, a structured qualitative synthesis was conducted. Studies were grouped and analyzed according to key treatment parameters, including radiation dose ranges, fractionation schemes, radiotherapy modality, and timing of postoperative radiotherapy.

## 3. Results

### 3.1. Data Collection

The initial search identified 653 articles. After removing duplicates, 421 studies were included for title or abstract review. Finally, 35 were selected for full-text appraisal, of which 22 met all the inclusion criteria and were included in this review. This systematic review included 22 studies comprising a total of more than 2219 patients. The study selection process is summarized in a PRISMA flow diagram (Figure 1).

After combining data from 22 publications published between January 2013 and July 2024, a total of 2219 patients treated for 2227 keloids were included in the analysis.

The methodological quality of the included studies ranged from moderate to high. Most studies achieved between six and nine stars on the Newcastle–Ottawa Scale, indicating generally acceptable to high methodological quality. Higher-quality studies were typically characterized by larger sample sizes, longer follow-up periods, and more clearly defined outcome assessment. However, several studies demonstrated limitations in comparability due to the lack of control for confounding variables and heterogeneity in treatment protocols, which may have influenced the reported outcomes.

The studies varied in terms of the size of the groups analyzed, ranging from studies involving a small number of patients [12] to large analyses involving 568 participants. Follow-up time after treatment ranged from 3 months to 160 months.

The analyzed study parameters included radiotherapy modality, total dose, fractionation schedule, timing of administration, recurrence rates and the incidence and severity of adverse effects, with attention paid to the time point at which these outcomes were evaluated.

Given the heterogeneity of the included studies, the results are presented using a structured qualitative approach, with subgroup analysis based on radiotherapy dose, fractionation, timing, and radiotherapy modality.

Detailed data are summarized in Table 2 and Table 3.

### 3.2. Recurrence Rate

Across the 22 analyzed publications, reported recurrence rates ranged from 1.6% to 55.2%. The lowest rate was observed in a study with a dose of 20 Gy in five fractions over 4 days using postoperative electron beam radiotherapy [18], whereas the highest was a total dose of 9 Gy using postoperative electron beam radiotherapy after wound closure with skin grafts [19]. In most studies, the recurrence rate was less than 30%. One study did not report the recurrence rate [20]. Based on categorized recurrence frequency, two studies reported rates up to 5%, eight studies reported rates between 5% and 10%, six studies documented recurrence rates between 10% and 20%, three studies between 20% and 30%, and another three studies reported rates exceeding 30%. Differences in recurrence rates were observed across studies reporting varying total radiation doses. When studies were grouped according to recurrence rate, lower recurrence rates were generally reported in studies using higher doses, whereas higher recurrence rates were more frequently observed in studies using lower doses. However, no formal statistical comparison was performed due to heterogeneity between studies. In one study, recurrence was 1.6% after a dose of 20 Gy and increased to 9.6% when doses below 20 Gy were used [Gy cohort with an odds ratio of 0.16 (confidence interval [CI] 0.036–0.75, *p* = 0.02) [18]. Recurrence rates varied across studies using different radiotherapy protocols. In one study, one group of patients received radiotherapy after surgery and skin grafting and the recurrence rate was as high as 55.2%, while the second group, after keloid incision, radiotherapy on the following day, keloid excision, skin grafting and further radiotherapy on the seventh day, had a recurrence rate of 16.7% [19]. Reported recurrence rates differed depending on the duration of follow-up. In one study, the recurrence rate increased from 32% at one year to 35% at five years [21]. In another study, the recurrence rate was 26.7% after one year, declined to 20.7% at three years and increased to 23.8% after prolonged follow-up [22].

### 3.3. Dose

A wide range of radiotherapy doses was reported across the 22 included studies, varying according to lesion location and the selected radiotherapy technique. The total doses ranged from 8 to 24 Gy and were delivered using both single and multiple fractions. The lowest dose of 8 Gy was used in two studies [23,24] and was administered in a single fraction. The highest total dose, 24 Gy, was administered as three fractions of 8 Gy each [25]. Radiotherapy with total doses of ≥20 Gy was applied in four studies, using schedules of three to five fractions [18,25,26,27].

The most frequently reported total dose range was 12 to 18 Gy applied in one to six fractions depending on the protocol. A total dose of 10 Gy or less was used in three studies, all of which used single-fraction radiotherapy [23,24,28].

### 3.4. Fractions

The 22 publications analyzed used various fractionation schedules, ranging from one to six fractions.

Single-fractionation schemes were used in four studies, with total doses ranging from 8 to 13 Gy, administered in a single session after surgery [23,28,29].

Two-fraction regimens are described in five publications [19,24,25,30,31]. Within these regimens, doses of 9 Gy × 2 were most used [19,30,31].

Three-fraction regimens were used in ten publications, with total doses ranging from 15 to 24 Gy [18,21,24,25,26,31,32,33,34,35]. The most used dose per fraction was 5–6 Gy.

Four-fraction schemes were also used in ten studies [18,20,21,22,26,27,32,36,37,38]. Total doses ranged from 12 to 20 Gy, with single fraction doses of 3 to 5 Gy. In most cases, fractions were administered daily. One study used a schedule with fractions administered every 2 days [38], and in one case, fractions were administered twice daily [22]. In one study, fractions were administered within 24 h, with 6 h intervals between each fraction [27].

Regimens involving more than four fractions (five to six fractions) were less common and were described in four publications [18,20,32,39]. The fractionation used was 2–4 Gy per fraction. The most commonly used number of fractions in the analyzed studies was three to four fractions, regardless of the radiotherapy technique used (external beam electron radiotherapy, HDR brachytherapy). Eight publications used different fractionation regimens within a single study group, where participants were treated according to different therapeutic protocols, differing in the number of fractions [18,20,21,24,25,26,31,32].

**Table 1:** Quality assessment of included studies using Newcastle–Ottawa Scale (NOS).

Study (Author, Year)	Selection	Comparability	Outcome	Total	Quality
---------------------	----------	--------------	--------	------	---------
Wang, 2014 [26]	⭐⭐⭐⭐	⭐⭐	⭐⭐⭐	9	High
Li, 2014 [19]	⭐⭐⭐	⭐	⭐⭐	6	Moderate
Kim, 2015 [20]	⭐⭐⭐	⭐	⭐⭐	6	Moderate
Lee, 2015 [32]	⭐⭐⭐⭐	⭐⭐	⭐⭐⭐	9	High
Shen, 2015 [30]	⭐⭐⭐⭐	⭐⭐	⭐⭐⭐	9	High
Jiang, 2016 [33]	⭐⭐⭐	⭐	⭐⭐	6	Moderate
Hafkamp, 2016 [29]	⭐⭐⭐	⭐	⭐⭐	6	Moderate
Carvajal, 2016 [21]	⭐⭐⭐	⭐	⭐⭐	6	Moderate
Bijlard, 2017 [31]	⭐⭐⭐⭐	⭐⭐	⭐⭐⭐	9	High
Renz, 2018 [18]	⭐⭐⭐⭐	⭐⭐	⭐⭐⭐	9	High
Sruthi, 2018 [24]	⭐⭐⭐	⭐	⭐⭐	6	Moderate
Sol, 2020 [23]	⭐⭐	⭐	⭐⭐	5	Moderate
Wang, 2020 [36]	⭐⭐⭐	⭐	⭐⭐	6	Moderate
Barragán, 2022 [37]	⭐⭐⭐⭐	⭐⭐	⭐⭐⭐	9	High
Hwang, 2022 [34]	⭐⭐⭐⭐	⭐⭐	⭐⭐⭐	9	High
Ha, 2022 [28]	⭐⭐	⭐	⭐⭐	5	Moderate
Bhattacharya, 2023 [27]	⭐⭐⭐⭐	⭐⭐	⭐⭐⭐	9	High
Katano, 2023 [35]	⭐⭐⭐	⭐	⭐⭐	6	Moderate
Fernandes, 2024 [25]	⭐⭐⭐	⭐	⭐⭐	6	Moderate
Ramelyte, 2024 [39]	⭐⭐⭐	⭐	⭐⭐	6	Moderate
Franzetti, 2024 [22]	⭐⭐⭐⭐	⭐⭐	⭐⭐⭐	9	High
Zhou, 2024 [38]	⭐⭐⭐⭐	⭐⭐	⭐⭐⭐	9	High

NOS—Newcastle–Ottawa Scale. The methodological quality and risk of bias of the included studies were assessed across three domains: Selection (maximum 4 stars), Comparability (maximum 2 stars), and Outcome (maximum 3 stars), with a total possible score of 9 stars. The number of stars awarded in each domain reflects the methodological rigor of the study. Studies scoring 7–9 stars were considered to have a low risk of bias, 4–6 stars a moderate risk of bias, and 0–3 stars a high risk of bias.

**Table 2:** Detailed data.

Authors	Year	Patients (n)	Therapy Method	Dose	Follow-Up (Months)	Recurrence Rate (%)	Complications
Lian-Zhao Wang [26]	2014	45	Postoperative electron beam radiotherapy, initiated 24–48 h after surgery	Total dose 15–20 Gy (5 Gy/day for 3–4 days)	24	2.2%	Skin exfoliation and pigmentation disorder (n = 8), resolved within 6 months Blister on treatment side (n = 1), resolved within 1 month
Wenbo Li [19]	2014	53	Postoperative electron beam radiotherapy Group 1 (n = 29) after skin grafting (1 and 7 day), Group 2 (n = 24) precut, incision, radiotherapy day after, excision, skin-grafting, radiotherapy after graft survival (1 and 10–14 day)	Total dose 18 Gy (9 Gy × 2),	12	55.2% (Group 1) 16.7% (Group 2)	Lack of aesthetic satisfaction (n = 14 in Group 1, n = 2 in Group 2)
Kyuhee Kim [20]	2015	28 (39 lesions)	Postoperative electron beam radiotherapy	Total dose 12–15 Gy 12 Gy for 4–5 days for Group 1 (13 lesions), 15 Gy for 4–5 days for Group 2 (25 lesions)	median 79.1 median 132 (range 132–160) for group 1 median 49.25 (range 15–124) for group 2	Not reported	Itching sensation (n = 6), pain (n = 5), no patients reported experiencing symptoms worse than those experienced before treatment
Sun Young Lee [32]	2015	30 (37 lesions)	Postoperative electron beam radiotherapy (6 MeV)	Total dose 12–18 Gy, administered every other day at 3–4 Gy, initiated within 24 h (24 lesions), 24–72 h (6 lesions), >72 h (7 lesions)	median 27.4 (range 9–51)	18.9%	Mild skin erythema/dermatitis grade 1 (8 lesions), transient hyperpigmentation (3 lesions)
Jie Shen [30]	2015	568 (834 lesions)	Postoperative high-energy electron beam radiotherapy (6–7 MeV) Group 1 (8 lesions) pre- and postoperative radiotherapy 48 h after operation, Group 2 (690 lesions) postoperative radiotherapy 48 h after, Group 3 (144 lesions) >48 h after surgery, Group 4 (36 lesions) 10–15 days after operation	Total dose 18 Gy (9 Gy × 2)	median 40 (range 12–160)	9.59%	Skin ulceration within the radiation field, unhealed wound, grafted skin necrosis, hyperpigmentation, teleangiectasia with depigmentation
Ping Jiang [33]	2016	24 (32 lesions)	Postoperative high-dose-rate brachytherapy, within 36 h after resection, first fraction within 6 h	Total dose 18 Gy (6 Gy × 3)	median 29.4 (range 7.9–72.4)	6%	Mild delay in the wound-healing process (n = 6), mild pigmentary abnormalities (n = 3), hyperpigmentation (n = 1), hypopigmentation (n = 2)
C.J.H. Hafkamp [29]	2016	24 (29 lesions)	Postoperative high-dose-rate brachytherapy	Total dose 13 Gy (13 Gy × 1)	median 53 (range 19–95)	24.10%	Infection (n = 1), chronic wound (n = 1), wound dehiscence (n = 1), hypopigmentation (n = 1)
Claudia C. Carvajal [21]	2016	63 (103 lesions)	Postoperative electron beam radiotherapy	Total dose 15–16 Gy (5 Gy × 3, 4 Gy × 4)	median 40.2 (range 12.3–85.6)	32% (after 1 year) 35% (after 5 years)	Bladder cancer, but field of radiotherapy did not include this organ (n = 1)
Eveline Bijlard [31]	2017	146 (238 lesions)	Postoperative high-dose-rate brachytherapy	Total dose 12–18 Gy (9 Gy × 2, 6 Gy × 3, 6 Gy × 2)	median 30.9 (2 × 9 Gy) 43.7 (3 × 6 Gy) 40.6 (2 × 6 Gy)	8.30%	Major complication (n = 29): severe wound dehiscence, severe infection, hyperpigmentation and hypopigmentation needing treatment, chronic wound > 3mo Minor complication (n = 103): wound dehiscence, infection, dermatitis grade 2, hyperpigmentation, hypopigmentation
Paul Renz [18]	2018	124 (250 lesions)	Postoperative electron beam radiotherapy	Total dose 12–20 Gy (4–5 Gy/day for 3–5 days)	median 40 (range 3–146)	5.6% (1.6–20 G), 9.6% (<20 Gy)	Breast cancer (n = 1) patient treated on 4 separate areas of the face; lung cancer (n = 1), several years after treatment, neuropathic pain in the right axillary region (n = 1), healing difficulties (n = 1)
K. Sruthi [24]	2018	30 (37 lesions)	Postoperative electron beam radiotherapy (6 MeV), single-fraction treatment in 91.9% of cases	Total dose 8–12 Gy for 1–3 days	median 32.67	16.20%	Postoperative wound dehiscence (n = 3)
Yuna Son et al. [23]	2020	12 (20 lesions)	Superficial X-ray radiotherapy	Single dose 8 Gy, 50 kV	median 24 (range 14–45)	6.25%	Mild hyperpigmentation in every patient, itching in 75% of patients
Yinmin Wang et al. [36]	2020	58 (58 lesions)	Electron beam radiotherapy (6 MeV), 12–16 Gy given in 4 fractions daily	6 MeV	median 22	8.60%	Hyperpigmentation in 5.17% patients
Victoria Vera Barragán [37]	2022	51 (61 lesions)	Postoperative high-dose-rate brachytherapy (HDR-BT) over 48 h, with 8 h intervals between fractions	12 Gy in 4 fractions	09.2011–10.2016 median 61, median 50.9 (16–96 range)	4.90%	—
Hwang, Na-Hyun [34]	2022	85 (136 lesions)	Postoperative electron beam radiotherapy (EBRT) based on CT mapping (3D dose distribution)	Total dose of 15–18 Gy provided in 3 equal fractions	01.2015–12.2020 median 71 months	7.40%	Each patient experienced grade 1 skin toxicity (follicular/matte erythema and dry desquamation). Late complications included wound dehiscence in the periumbilical area in 1 case, and hypopigmentation or hyperpigmentation in 10 cases
Boram Ha [28]	2022	16	Postoperative electron beam radiotherapy within 8 h after surgery	Total dose of 10 Gy in a single fraction	median 12 months range 10–14 months	18.75%	2 cases of hyperpigmentation (resolved within one year), 2 cases of acute wound dehiscence, and 1 case of acute radiodermatitis (resolved after 3–4 weeks)
Neela Bhattacharya [27]	2023	50	Postoperative electron radiotherapy and HDR brachytherapy within 24 h after surgery, with 6-h intervals between fractions	20 Gy in 4 fractions of 5 Gy each, with 6-h intervals between fractions	2004–2021 minimum 18 months	6%	In 5 patients, delayed wound healing in the 2nd week required delayed suture removal; 4 patients developed hyperemia, and 1 patient had facial edema. At week 4, 5 patients developed hypertrophic scars, which resolved with conservative treatment; in 2 patients, the scars widened. After 6 months: 1 patient experienced pain due to a suture granuloma, and 1 patient developed a nodule at 12 months, which progressed to a hypertrophic scar. In 4 patients, scars widened, with recurrence in 2 of these cases
Katano Atsuto [35]	2023	81 (94 lesions)	Postoperative electron beam radiotherapy	6 MeV on the day of surgery or the next day, 15 Gy in 3 fractions	median 17.8 range 3–93.4	13.60%	Mild and moderate adverse events including dermatitis
J. Fernandes, D. Liao [25]	2024	41 (70 lesions)	Postoperative electron beam radiotherapy, the remainder treated with photons and orthovoltage (55 keloids). Definitive radiotherapy: 3 fractions (15 keloids) on days 0, 7, and 21	16 Gy in 2 fractions over 4 days (82.8% of cases), Definitive radiotherapy: 24 Gy in 3 fractions	01.2014–09.2020 median	18.80%	Acute complications: 18.6% experienced grade 1 or 2 dermatitis, and 1 case of grade 3 dermatitis. Late complications: grade 1 hyperpigmentation in 38.9% of patients; other late complications were rare. No malignancies were observed
Egle Ramelyte [39]	2024	90 (104 lesions)	Postoperative PER radiotherapy (photon and electron beams) each, started 24–48 h after keloid excision	Total dose of 12 Gy administered in 6 fractions of 2 Gy	24 months	50%	Mild local hyperpigmentation occurred in 34.4% of patients. In 71% of these cases, it completely resolved within 2 years. It was most common in patients of Hispanic origin, with skin types IV and V, on the trunk and upper limbs, and was almost absent on the ear and head
Jessica Franzetti [22]	2024	102 (135 lesions)	Postoperative high-dose-rate interventional radiotherapy (POIRT) HDR-IRT (brachytherapy) twice daily, started within 4–6 h after surgery	Total dose of 12 Gy in 4 fractions (3 Gy per fraction)	01.2004–07.2020 median 64 months range 25–103	26.7% after 12 months 20.7%, after 36 months 23.8% after longer period	Acute complications (within 3 months)—erythema and pruritus in 17.8% and 5.9% of patients, respectively. Late complications (after 3 months)—fibrosis 13.2%, dyschromia 5.3%, wound dehiscence 3.5%, telangiectasia 0.9%
Wei Zhou [38]	2024	498	Postoperative electron beam radiotherapy with 4 MeV energy, every other day, started 24 h after the procedure	16 Gy in 4 fractions	01.2010–12.2017 median 68,1 range 42.6–129.9	26.50%	In 2 patients, wound infection occurred; in 1 patient, a fibroblastoma developed in the area that received radiotherapy near the pubic region. Pain or pruritus was reported in 16.3% of patients

Legend (abbreviations): BT—brachytherapy; EBRT—external beam radiotherapy; Gy—Gray (unit of radiation dose); HDR—high dose rate; MeV—mega-electron volt; POIRT—postoperative interventional radiotherapy.

**Table 3:** Detailed data.

Authors	Year	Patients (n)	Sex	Lesion Location	Age (Years)	Etiology	Medical History
Lian-Zhao Wang [26]	2014	45	30 females 15 males	Chest	median 32 (range 18–49)	Unprovoked (n = 4) Scratch (n = 3) Folliculitis (n = 32) Mosquito bites (n = 4) Surgery (n = 2)	Prior history of keloid treatment range 1–12 years
Wenbo Li [19]	2014	53	21 female 32 male	Chest	median age 23 ± 5 in Group 1 median age 21 ± 6 in Group 2	Not reported	Not reported
Kyuhee Kim [20]	2015	28 (39 lesions)	Not reported	Ear	Not reported	Not reported	Not reported
Sun Young Lee [32]	2015	30 (37 lesions)	23 females 7 males	Ear (n = 28) Shoulder (n = 4) Chest (n = 3) Abdomen (n = 2)	median 23.8 (range 11–66)	Piercing (n = 24) Trauma (n = 5) Surgery (n = 3) Acne (n = 2) vaccination scar (n = 3)	Not reported
Jie Shen [30]	2015	568 (834 lesions)	465 females 103 males	Ear (n = 239) Back (n = 36) Face (n = 19) Neck (n = 57) Shoulder (n = 56) Chest (n = 206) Abdomen (n = 120) Upper limbs (n = 68) Lower limbs (n = 33)	median 29 (range 5–80)	Not reported	Patients with prior keloid treatment were excluded
Ping Jiang [33]	2015	24 (32 lesions)	15 females 9 males	Ear (n = 10) Chest (n = 11) Other (n = 11)	median 44 ± 18.3 (range 20–80)	Acne Surgery Piercing Injury	Prior lesion treatment without sustained remission
C.J.H. Hafkamp [29]	2017	24 (29 lesions)	12 females 12 males	Ear (n = 14) Head (n = 2) Abdomen (n = 5) Neck (n = 1) Shoulder (n = 2) Chest (n = 5)	median 31 (range 15–64)		No prior treatment
Claudia C. Carvajal [21]	2016	63 (103 lesions)	29 females 34 males	Ear (n = 18) Face (n = 6) Neck (n = 10) Shoulder (n = 16) Chest (n = 22) Abdomen (n = 16) Upper limbs (n = 11) Lower limbs (n = 1)	median 30 (range 13–77)	Piercing (n = 13) Surgery (n = 36) Vaccination (n = 6) Acne (n = 10) Trauma (n = 18) Burn (n = 14)	Prior treatment with corticosteroids
Eveline Bijlard [31]	2018	146 (238 lesions)	Group 1: 19 females 24 males Group 2: 28 females 26 males Group 3: 30 females 19 males	Chest (n = 106) Ear (n = 78) Other (n = 54)	Group 1: median 36.4 Group 2: median 32.9 Group 3; median	Not reported	Not reported
Paul Renz [18]	2018	124 (250 lesions)	87 females 37 males	Ear (n = 86) Head and neck (n = 70) Chest (n = 83) Limb (n = 11)	median 34 (range 14–84)	Not reported	Patients with prior adverse effects to treatment were excluded
K. Sruthi [24]	2018	30 (37 lesions)	Not reported	Ear (n = 8) Head and neck (n = 8) Chest (n = 12) Limb (n = 6) Haunch (n = 4) Shoulder and back (n = 11)	median 37 (range 21–77)	Not reported	Not reported
Yuna Son et al. [23]	2020	15 (20 lesions)	7 females 8 males	Back (n = 1) Head and neck (n = 9) Chest (n = 4) Pubis (n = 1) Shoulder (n = 1) Abdomen (n = 1) Ear (n = 3)	Not reported	Not reported	Not reported
Yinmin Wang et al. [36]	2020	58 (58 lesions)	28 females 30 males	Ear (n = 8) Head and neck (n = 8) Abdomen (n = 9) Limb (n = 6) Chest (n = 12) Shoulder and back (n = 11) Haunch (n = 4)	median 33 (range 21–76)	Not reported	Patients with only one lesion, diagnosed as keloid and reacts to surgery and radiotherapy
Victoria Vera Barragán [37]	2022	51 (61 lesions)	25 females 26 males	Ear (n = 19) Limb (n = 7) Chest (n = 25) Back (n = 2) Other (n = 8)	median 46 (range 20–89)	Surgery (n = 18) Trauma (n = 19) Acne (n = 22) Other (n = 2)	Not reported
Hwang, Na-Hyun [34]	2022	85 (136 lesions)	63 females 22 males	Ear (n = 92) Head and neck (9) Trunk (16) Limb (9)	median 25 (range 15–77)	Piercing (n = 89) Surgery (n = 26) Burn (n = 2) Acne (n = 1) Vaccination (n = 1) Tattoo (n = 1) Unknown (n = 16)	Prior treatment and family history (n = 3) Family history in first degree relative (n = 2)
Boram Ha [28]	2022	16	16 females 9 males	Chest	median 32	Acne (n = 8) Sebum (n = 2) Surgery (n = 3) Burn (n = 1)	Not reported
Neela Bhattacharya [27]	2023	50	40 females 10 males	Ear (n = 24) Face (n = 7) Neck (n = 4) Shoulder (n = 3) Upper limb (n = 2) Lower limb (n = 2) Abdomen (n = 2) Breast (n = 1) Back (n = 1)	mostly between 21–30	Piercing (n = 18 Infection (n = 10) Trauma (n = 17) Allergic dermatitis (n = 2) Surgery (n = 3)	Prior history of keloid occurrence ranged from 6 months to 18 years, history of various prior treatment in different patients
Katano Atsuto [35]	2023	81 (94 lesions)	59 females 22 males	Ear (n = 20) Chest (n = 19) Neck (n = 2) Shoulder (n = 5) Limb (n = 5) Abdomen (n = 32) Breast (n = 7) Suprapubic (n = 4)	median 47 (range 20–80)	Not reported	Patients who had prior radiotherapy have been excluded
J. Fernandes, D. Liao [25]	2024	41 (70 lesions)	24 females 17 males	Ear (n = 21) Head and neck (n = 10) Chest (n = 29) Arm (n = 1) Shoulder and axilla (n = 7) Vulva (n = 2)	median 37 (range 19–80)	Acne (n = 7) Trauma (n = 4) Piercing (n = 17) Ingrown hair (n = 1) Post-varicella (n = 1) Burn (n = 1) Iatrogenic (n = 9) Post-shaving (n = 1) Not specified (n = 27)	Prior treatment: Steroid injections (n = 32) Single surgical resection (n = 9) Multiple surgical resection (n = 7) None (n = 27)
Egle Ramelyte [39]	2024	90 (104 lesions)	53 females 37 males	Ear (n = 22) Head and neck (n = 15) Trunk (n = 51) Arm (n = 1) Upper limb (n = 16)	median 37 (range 13–78)	Piercing (n = 21) Spontaneous (n = 8) Surgery (n = 52) Trauma (n = 19) Unknown (n = 4)	Prior treatment reported (n = 49) No prior treatment reported (n = 41)
Jessica Franzetti [22]	2024	102 (135 lesions)	83 females 19 males	Ear (n = 18 Abdomen (n = 18) Chest (n = 80) Limb (n = 9) Neck (n = 7) Other (n = 3)	median 43 (range 16–76)	Surgery (n = 100) Spontaneous (n = 10) Other (n = 25)	Not reported
Wei Zhou [38]	2024	498	372 females 126 males	Ear (n = 298) Head and neck (n = 44) Chest (n = 63) Abdomen (n = 32) Perineum (n = 37) Limb and back (n = 24)	median 25 (range 4–77)	Piercing (n = 298) Scar (n = 44) Trauma (n = 56) Other (n = 37)	Prior treatment: Surgery alone reported (n = 337) Compression and surgery (n = 18) Corticosteroid and surgery (n = 26) Silicone and surgery (n = 82) Compression combined with silicone, corticosteroids, surgery (n = 35)

### 3.5. Timing of Radiotherapy Administration

In 14 studies describing a total of 1261 patients, the first dose of radiotherapy was most frequently applied within 24 h following surgical excision. In six additional articles, involving 893 patients, radiotherapy was administered between 24 and 48 h after surgery. In the remaining paper with 12 treated patients, this method was initiated after more than 48 h [23]. Ref. [19] presented a novel precut and preradiotherapy regime, involving a preliminary incision to the subcutaneous layer around the keloid margin, with radiotherapy performed the following day. The keloid was subsequently excised, the wound closed with a skin graft, and a second dose of radiotherapy was administered after successful graft take on day 10–14. This variability does not allow for identification of an optimal timing for postoperative radiotherapy. However, earlier treatment was more commonly reported across studies.

### 3.6. Methods of Radiotherapy

Radiotherapy can be provided in many ways, depending on clinical conditions and the individual situation of the patient. When analyzing the use of radiotherapy in treating patients with keloids, the most significant methods are postoperative electron beam radiotherapy, postoperative high-dose-rate radiotherapy, postoperative low-dose-rate radiotherapy, and superficial X-ray radiotherapy.

X-ray radiotherapy, which uses photons to treat affected tissue, is less commonly used in keloid treatment and was described in a limited group of 12 patients with 20 lesions. The recurrence rate was 6.25%, and only mild adverse effects were observed [23].

Postoperative photon and electron radiotherapy was used in one group of 90 patients and 104 lesions. This group experienced mild skin hyperpigmentation in 34.4%. What emerges as most vital is the recurrence rate of 50% [39].

Brachytherapy is a technique that delivers localized radiation to a targeted area through a specifically designed applicator placed directly within or in close proximity to the treatment site, most commonly following surgical excision. This approach allows for the administration of relatively high radiation doses with high spatial precision. In the included studies, 397 patients with 545 lesions were treated with brachytherapy. Among them, 295 patients with 410 lesions received conventional high-dose-rate (HDR) brachytherapy [29,31,33,37], while 102 patients with 135 lesions were treated with postoperative high-dose-rate interventional radiotherapy [22].

Although interventional radiotherapy appeared to be associated with higher recurrence rates compared to conventional HDR brachytherapy, a formal statistical comparison between different brachytherapy regimens was not feasible due to substantial heterogeneity in radiation dose, fractionation schedules, follow-up duration, and outcome definitions across studies. Reported recurrence rates for brachytherapy ranged from approximately 4.9% [37] and 6% [33] to 24.1% [29] and over 20% in longer follow-up analyses [22]. However, these differences should be interpreted with caution, as variability in study design, patient population, and follow-up period may significantly influence outcomes. Therefore, no statistically valid conclusions can be drawn regarding the superiority of one brachytherapy approach over another, and further prospective studies with standardized protocols are required.

Another option, crucial when treating keloids, is electron beam radiotherapy. Electron beam radiotherapy is a method which uses electrons externally to destroy cells on the superficial surface of the body. Electrons have limitations associated with penetration into deeper layers; therefore, this prevents the formation of pathological masses in deeper areas. In total, 1729 patients were treated with electron beam radiotherapy, 50 of them treated with high-dose brachytherapy and electron beam radiotherapy simultaneously. When these two methods were used together, recurrence rates of approximately 6% were reported [27]. In the same study, no severe adverse effects were observed. Patients with surgical incision before electron beam radiotherapy with a skin flap (n = 24) had a significantly lower recurrence rate (16.7%) compared to those treated with the postoperative technique (55.2%) [19].

The method which is applied most frequently, and which allows us to analyze the widest range of patients, is electron beam radiotherapy. Combining this approach with other treatment methods was associated with lower reported recurrence rates, without a clear increase in reported adverse effects [27].

## 4. Discussion

This systematic review summarizes the current evidence supporting the effectiveness of combining surgery with radiotherapy for the treatment of keloids. The combination of these modalities is currently regarded as an effective adjuvant approach that may reduce the risk of recurrence compared with surgery alone. However, a meta-analysis could not be performed due to the substantial heterogeneity of the available data, which constitutes a limitation of this study. The methods of postoperative radiotherapy currently in use are electron beam radiotherapy and brachytherapy. A recent meta-analysis by Seth et al. (2026) on post-excisional radiotherapy for keloids demonstrated that postoperative radiotherapy significantly reduces keloid recurrence; however, no clear superiority of one radiotherapy modality over another was established due to heterogeneity among studies [12]. Brachytherapy involves placing radioactive material within the treatment site using a catheter, while electron beam radiotherapy and X-ray radiotherapy use an external radiation source.

Lee et al. and Bijlard et al. used radiotherapy dose regimens ranging from 12 to 18 Gy administered within 48 h after surgical excision of the lesion, resulting in recurrence rates of 8.3% and 18.9%, respectively [31,32].

A recurrence rate of 6% was achieved by Jiang et al. and Bhattacharya et al., who used a dose of 18 Gy administered in three fractions and a total dose of 20 Gy administered in four fractions, respectively [27,33]. A similar recurrence rate of 6.25% was reported by Son et al. who used a single dose of 8 Gy administered in one fraction using the X-ray radiotherapy method [23]. The apparent discrepancies in recurrence rates across studies may reflect differences in follow-up duration, radiotherapy protocol, and patient- and lesion-related factors, rather than true inconsistencies in treatment effectiveness.

Wang et al., administering doses of 15 to 20 Gy in three to four fractions, obtained recurrence rates of 2.2% [26]. Similarly, very low recurrence rates have been reported in studies applying higher total doses, suggesting a possible dose–response relationship.

A potential dose–response relationship between radiation dose and recurrence rate was observed across the included studies, with lower recurrence rates generally reported in studies using higher total doses. However, this observation is based on indirect comparisons between heterogeneous studies and was not supported by formal statistical analysis or adjustment for potential confounding factors, such as lesion location, radiotherapy technique, or patient characteristics. Therefore, this relationship should be interpreted with caution and considered a trend rather than definitive evidence.

Ramelyte et al. [39] used a relatively low dose of 12 Gy administered in six fractions of 2 Gy each and obtained recurrence rates of 50% 39. Barragán et al. report different results—when using the same total dose of 12 Gy spread over four fractions, a recurrence rate of 4.9% was observed [37]. These discrepancies may be attributable not only to differences in fractionation schemes, but also to variations in radiotherapy technique, lesion location, patient characteristics, and follow-up duration. The radiotherapy doses reported in the included studies are not standardized, and a more in-depth analysis would be required to determine a potential relationship between dose and treatment timing. Such an assessment would be more feasible in the context of well-designed prospective studies. At present, the available evidence suggests that radiotherapy is effective in reducing keloid recurrence; however, it remains unclear which specific modality or dosing regimen is optimal. Variability in follow-up duration represents an important source of heterogeneity that directly impacts the interpretation of recurrence rates. Studies included in this review reported follow-up periods ranging from a few months to several years, which may lead to substantial differences in observed recurrence rates. Short follow-up durations may underestimate recurrence due to delayed manifestation of keloid regrowth, whereas longer follow-up increases the likelihood of detecting late recurrences. Consequently, differences in follow-up length should be considered when comparing outcomes across studies and may partly explain the variability in reported recurrence rates.

Some studies recommend starting radiotherapy within a few hours of keloid excision surgery, while others indicate that it should be performed within 48 h of surgery. In the study by Zhou et al. [38], 16 Gy was administered in four fractions starting 24 h after surgery, and a recurrence rate of 26.5% was obtained. Similar recurrence rates were obtained by Franzetti et al., using a total fractionated dose of 12 Gy in 3 Gy fractions, with the first radiotherapy dose administered 4 to 6 h after keloid excision [22]. These findings could suggest that, while early initiation of radiotherapy is generally preferred, the timing alone may not be the decisive factor influencing recurrence rates. The radiotherapy modality and total dose may play a more substantial role.

The use of radiotherapy is associated with a theoretical risk of carcinogenesis. In the two studies considered, three cases of secondary malignant tumors were reported. In Carvajal et al., one patient was diagnosed with bladder cancer, although this organ was not within the radiotherapy field [21]. A similar situation was reported by Renz et al., where two cases of malignant tumors were documented; however, the affected organs were also not located within areas that received radiotherapy [18]. Therefore, a direct causal relationship between postoperative radiotherapy for keloids and secondary malignancy development could not be established based on the available data. The long-term safety of adjuvant radiotherapy remains an important consideration. Although only a few cases of secondary malignancies were reported, a direct causal relationship with radiotherapy could not be established. It should be noted that the available evidence is limited by relatively short follow-up periods in many studies, as well as the retrospective design and lack of systematic long-term surveillance. These limitations restrict the ability to draw firm conclusions regarding long-term oncological safety.

The use of radiotherapy may increase the risk of complications associated with the local application of radiotherapy.

The most common complications observed in patients are hypopigmentation, hyperpigmentation, skin thinning, itching, delayed wound healing, telangiectasia, adverse aesthetic effects, and the recurrence of pathological changes. Most reported adverse effects were mild to moderate in severity and localized to the treated area.

Despite its proven effectiveness when combined with surgery, radiotherapy has certain limitations that should be considered. Due to its dynamic development, there is a lack of standardized guidelines regarding the preferred radiotherapy technique, total dose, and fractionation schedule. This lack of standardization limits the routine and reproducible application of radiotherapy across larger patient populations. The variability in keloid morphology and anatomical location further complicates the selection of an optimal dose and the achievement of homogeneous dose distribution within the treatment field.

Another limitation is the ongoing lack of consensus regarding the optimal radiotherapy dose and the most appropriate timing of postoperative radiotherapy initiation. The need for individualized treatment planning, combined with heterogeneous clinical protocols, makes it difficult to define a universal therapeutic window in which radiotherapy would yield uniformly effective outcomes.

Our findings are consistent with current clinical practice and routinely applied treatment approaches. However, there is a lack of standardized prospective studies on the basis of which treatment protocols and clinical guidelines could be established.

## 5. Conclusions

The analyzed parameters included radiotherapy modality, total dose, fractionation schedule, timing of administration, recurrence rates and the incidence of adverse effects. Due to the heterogeneity of the included studies, a structured qualitative approach with subgroup analyses was applied. These subgroup analyses were based on radiotherapy dose, fractionation, timing, and modality, allowing for a more nuanced interpretation of the results. Overall, the findings suggest that the combination of surgery and postoperative radiotherapy may be associated with reduced recurrence rates compared with surgery alone. However, the variability in treatment protocol and study design makes direct comparisons challenging, and the observed differences in outcomes could be influenced by multiple factors, including lesion characteristics, radiotherapy technique, and patient-related variables.

Consequently, while adjuvant radiotherapy appears to be effective and generally safe, further high-quality prospective studies with long-term follow-up are required to better define optimal treatment parameters and to fully assess long-term risks.

## 6. Strengths and Limitations of Study

A major strength of this study is that it provides, to the best of our knowledge, the most current and comprehensive synthesis of the available evidence on adjuvant radiotherapy in keloid management. By integrating findings across a wide range of studies, this review offers a thorough and clinically meaningful overview of contemporary treatment approaches, thereby contributing valuable insight to the existing literature.

The findings of this review should be interpreted in light of several limitations related to the quality of the included studies. Although most studies were rated as moderate- to high-quality based on the Newcastle–Ottawa Scale, a moderate risk of bias was identified in a substantial proportion of studies. This was primarily related to the retrospective design, lack of control groups, and limited adjustment for potential confounding factors.

A major limitation of the present review is the substantial heterogeneity across the included studies. This variability encompasses differences in radiotherapy techniques, dose regimens, fractionation schedules, timing of radiotherapy initiation, and duration of follow-up. Additionally, inconsistencies in outcome reporting, particularly in the definition and timing of recurrence assessment, further limit direct comparisons between studies. This heterogeneity not only precluded the performance of a meta-analysis, but also impacted the generalizability of the findings. Therefore, the results should be interpreted with caution, and emphasis should be placed on trends observed across studies rather than direct quantitative comparisons.

## 7. Future Directions

Further high-quality, prospective studies with standardized treatment protocols are needed to strengthen the evidence base.

## Data Availability

Not applicable.
